# Pegfilgrastim prophylaxis is associated with a lower risk of hospitalization of cancer patients than filgrastim prophylaxis: a retrospective United States claims analysis of granulocyte colony-stimulating factors (G-CSF)

**DOI:** 10.1186/1471-2407-13-11

**Published:** 2013-01-08

**Authors:** Arash Naeim, Henry J Henk, Laura Becker, Victoria Chia, Sejal Badre, Xiaoyan Li, Robert Deeter

**Affiliations:** 1 Department of Medicine, Division of Hematology-Oncology, Jonsson Comprehensive Cancer Center, University of California, Los Angeles, CA, USA; 2 OptumInsight, Health Economics and Outcomes, 12125 Technology Drive, Eden Prairie, MN, 55344, USA; 3 Center for Observational Research, Amgen Inc., One Amgen Center Drive, Thousand Oaks, CA, 91320, USA; 4 Global Biostatistical Science, Amgen Inc., One Amgen Center Drive, Thousand Oaks, CA, 91320, USA; 5 Global Health Economics, Amgen Inc., One Amgen Center Drive, Thousand Oaks, CA, 91320, USA

## Abstract

**Background:**

Myelosuppressive chemotherapy can lead to dose-limiting febrile neutropenia. Prophylactic use of recombinant human G-CSF such as daily filgrastim and once-per-cycle pegfilgrastim may reduce the incidence of febrile neutropenia. This comparative study examined the effect of pegfilgrastim versus daily filgrastim on the risk of hospitalization.

**Methods:**

This retrospective United States claims analysis utilized 2004–2009 data for filgrastim- and pegfilgrastim-treated patients receiving chemotherapy for non-Hodgkin’s lymphoma (NHL) or breast, lung, ovarian, or colorectal cancers. Cycles in which pegfilgrastim or filgrastim was administered within 5 days from initiation of chemotherapy (considered to represent prophylaxis) were pooled for analysis. Neutropenia-related hospitalization and other healthcare encounters were defined with a “narrow” criterion for claims with an ICD-9 code for neutropenia and with a “broad” criterion for claims with an ICD-9 code for neutropenia, fever, or infection. Odds ratios (OR) for hospitalization and 95% confidence intervals (CI) were estimated by generalized estimating equation (GEE) models and adjusted for patient, tumor, and treatment characteristics. Per-cycle healthcare utilization and costs were examined for cycles with pegfilgrastim or filgrastim prophylaxis.

**Results:**

We identified 3,535 patients receiving G-CSF prophylaxis, representing 12,056 chemotherapy cycles (11,683 pegfilgrastim, 373 filgrastim). The mean duration of filgrastim prophylaxis in the sample was 4.8 days. The mean duration of pegfilgrastim prophylaxis in the sample was 1.0 day, consistent with the recommended dosage of pegfilgrastim - a single injection once per chemotherapy cycle. Cycles with prophylactic pegfilgrastim were associated with a decreased risk of neutropenia-related hospitalization (narrow definition: OR = 0.43, 95% CI: 0.16–1.13; broad definition: OR = 0.38, 95% CI: 0.24–0.59) and all-cause hospitalization (OR = 0.50, 95% CI: 0.35–0.72) versus cycles with prophylactic filgrastim. For neutropenia-related utilization by setting of care, there were more ambulatory visits and hospitalizations per cycle associated with filgrastim prophylaxis than with pegfilgrastim prophylaxis. Mean per-cycle neutropenia-related costs were also higher with prophylactic filgrastim than with prophylactic pegfilgrastim.

**Conclusions:**

In this comparative effectiveness study, pegfilgrastim prophylaxis was associated with a reduced risk of neutropenia-related or all-cause hospitalization relative to filgrastim prophylaxis.

## Background

Patients receiving myelosuppressive chemotherapy are at risk of developing febrile neutropenia, a major dose-limiting toxicity of systemic chemotherapy associated with hospitalization, use of intravenous antibiotics, and significant morbidity, mortality, and costs [1-10]. Recombinant human granulocyte colony-stimulating factor (G-CSF; filgrastim and pegfilgrastim) is indicated to reduce the incidence of infection in patients with non-myeloid malignancies receiving myelosuppressive chemotherapy [2,4,5]. Guidelines recommend the prophylactic use of G-CSF in patients receiving chemotherapy regimens that have a 20% or greater risk of febrile neutropenia [11,12]. In addition, patients receiving chemotherapy regimens associated with a febrile neutropenia risk below 20% may have a total combined risk above 20% if they have additional risk factors, such as comorbid conditions or advanced age. Thus, these patients may benefit from prophylactic G-CSF use as well [11-13].

Filgrastim is recommended to be administered at a recommended starting dose of 5 mcg/kg per day until neutrophil recovery occurs after the expected chemotherapy-induced nadir. Its short circulating half-life (~3.5 hours) necessitates that it be given daily. The efficacy of filgrastim depends on the number of days it is administered. Randomized comparative clinical trials have shown that patients treated with a mean of 10–11 filgrastim doses per chemotherapy cycle reported a similar decrease in the duration of severe neutropenia as patients treated with pegfilgrastim once per chemotherapy cycle [14-18]. In clinical practice, filgrastim is often administered for fewer than 10–11 days and may be associated with reduced efficacy [19]. A chart review study found that the risk of hospitalization was approximately one-third higher with filgrastim use compared to pegfilgrastim use [20]. An additional retrospective observational study of United States claims databases showed that prophylactic use of pegfilgrastim was associated with a one-third to two-thirds reduction in the risk of hospitalization for febrile neutropenia relative to the risk in patients who received filgrastim prophylaxis [21]. Two more recent studies on comparative effectiveness of G-CSF prophylaxis reported similar findings based on United States claims data [22,23].

Neutropenic complications following chemotherapy contribute significantly to the costs of cancer care. During 1989–2007, the number of neutropenia-related hospitalizations among cancer patients in the United States was estimated to be approximately 57,000–103,000 per year [10]. In a study using 1995–2000 data, the average cost per hospitalization due to febrile neutropenia was reported to be $12,372 for breast cancer patients, $18,437 for lymphoma patients, and $38,583 for leukemia patients [3]. Another study using 2005–2008 data found that mean hospitalization costs were $18,042 for cancer patients with neutropenia, $22,839 for those with neutropenia plus infection or fever, and $27,587 for those with neutropenia plus infection [9]. Clearly, neutropenic complications in patients receiving chemotherapy pose a significant medical and financial burden.

The primary objective of the current study was to determine whether a difference in the risk of neutropenia-related and all-cause hospitalization between chemotherapy cycles associated with filgrastim prophylaxis and cycles with pegfilgrastim prophylaxis. This study from a United States claims database included data from January 2004 through February 2009 regarding filgrastim and pegfilgrastim administration patterns and related clinical outcomes. Additionally, economic data in the form of comparative healthcare utilization and costs results are described.

## Methods

### Study design

This study was a retrospective United States claims analysis using data from health plans affiliated with OptumInsight (formally Ingenix). This national database contains both medical and pharmacy claims with linked enrollment information data beginning in 1993. As of 2008, medical and pharmacy benefit coverage information was available for over 14 million individuals.

All patient-identifying information was either encrypted or removed from the study database prior to its release to the study investigators. The study database does not contain any Protected Health Information and is fully compliant with the Health Insurance Portability and Accountability Act (HIPAA) of 1996 and federal guidance on Public Welfare and the Protection of Human Subjects [24,25]. As per the Code of Federal Regulations, Institutional Review Board review and approval is not needed for a study of this nature, as “…subjects cannot be identified, directly or through identifiers linked to the subjects…” (45 CFR 46 §46.101). Use of this fully de-identified and HIPAA-compliant study database for health services research is therefore in full compliance with the Helsinki Declaration [26].

Outcomes, including administration patterns, neutropenia-related and all-cause hospitalization rates, and utilization and cost data, were obtained for both filgrastim- and pegfilgrastim-treated patients receiving chemotherapy for non-Hodgkin’s lymphoma (NHL), breast cancer, lung cancer, ovarian cancer, or colorectal cancer.

Patients with chemotherapy medical claims between January 1, 2005 and February 28, 2009 were studied. The year prior to the index date was used to determine whether patients met inclusion/exclusion criteria and to provide demographic and patient characteristic data. The date of the first chemotherapy claim of an eligible patient was deemed the index date. Patients were included if they had two or more medical claims ( ≥ 7 days apart) with ICD-9 code(s) for NHL, breast cancer, lung cancer, ovarian cancer, or colorectal cancer from 30 days prior to the index date up to 30 days after the index date, and one or more claim for filgrastim or pegfilgrastim (not both) during the chemotherapy course. Claims from laboratories, diagnostic testing centers, or any diagnostic tests were not considered when identifying cancer patients, as well as claims with “rule-out” codes (CPT-4 codes 36400–36425, 70010–76999, 7800–78799, 80000–89999; HCPCS codes S9529, G0001). Patients were excluded if they had less than 1 year of continuous eligibility preceding the index date; any claim for chemotherapy during the 1 year prior to the index date; one or more medical claims for bone marrow or stem cell transplant; claims indicating sargramostim use; claims for services provided in skilled nursing facility or hospice services; or if they had codes for more than one type of primary cancer. Patients with metastatic disease were not specifically excluded.

Besides tumor type and use of pegfilgrastim/filgrastim, data collected also included demographic characteristics, comorbid conditions, cancer treatment history, and chemotherapy agents received in each chemotherapy cycle. The first eligible chemotherapy course for each patient after January 1, 2005 was used in this analysis. Each chemotherapy course may include several cycles. The first chemotherapy course began on the index date and ended with any of the following, whichever came first: 1) the absence of any chemotherapy claims within the 60 days after a chemotherapy claim (ie, chemotherapy gap); 2) the end of insurance eligibility or study period; or 3) the initiation of radiation therapy. Chemotherapy cycles in the course were defined to identify unique cycles of interest, and were excluded if two chemotherapy claims had less than 20 days between them or if there were chemotherapy claims from days 7–19 of a cycle [21]. Chemotherapy cycle length was restricted to ensure a more homogeneous treatment population consistent with the labeled indications of both filgrastim and pegfilgrastim, as pegfilgrastim is not indicated to support weekly or every-two-week chemotherapy cycles.

For both filgrastim and pegfilgrastim, use was categorized as prophylactic (ie, initiated on or before day 5 of a cycle) or delayed (initiated after day 5 of a cycle) [19,21]. The analysis sample of the study only included cycles in which G-CSF was used prophylactically. We used G-CSF initiation during days 1–5 after the start of the cycle to identify prophylaxis for two reasons: most chemotherapy regimens are administered over a 1- to 3-day period, and G-CSF prophylaxis is recommended to be initiated within 24 to 72 hours after chemotherapy (ie, generally by the end of cycle day 5); febrile neutropenia rarely occurs within the first 5 days of a cycle, and thus G-CSF use during this period would almost certainly constitute prophylaxis rather than treatment for febrile neutropenia. Cycles associated with delayed G-CSF use were excluded from the analysis. Neutropenia-related hospitalization and other healthcare encounters were defined with both a “narrow” criterion for claims with neutropenia (288), and with a “broad” criterion for claims with neutropenia or fever of unknown origin (ICD-9: 780.6) or infection (codes are listed in the Additional file 1). The primary study endpoints were whether all-cause hospitalization and neutropenia-related hospitalization (ie, hospitalizations for which there were neutropenia codes as defined above) occurred within a chemotherapy cycle associated with filgrastim or pegfilgrastim prophylaxis.

### Utilization and cost data

Healthcare utilization and costs were calculated for each cycle. Total all-cause costs were calculated as the costs associated with all medical and pharmacy claims during the cycle. Physician fees, chemotherapy costs, and G-CSF costs were all included in the total all-cause cost measure. Additionally, all-cause utilization and costs were calculated separately for emergency room (ER) visits, hospitalizations, and ambulatory care visits (outpatient hospital and office-based visits). Hospitalization length of stay was another examined utilization outcome. Drugs delivered as part of medical benefits (eg, administered chemotherapy) were included in the costs for each setting of care: ER, hospital inpatient, and ambulatory care. Retail pharmacy costs were calculated as the cost for all prescription claims and considered as a component of total costs in the ambulatory care setting. Neutropenia-related utilization and costs were calculated from claims associated with neutropenia. The costs represented the reimbursed amount paid by the patient and insurer. Only direct costs for services covered under the patient’s insurance benefit were included in this study.

### Statistical analyses

Descriptive statistics regarding patient characteristics and cycle characteristics (in terms of chemotherapy agents received and G-CSF administration patterns) were calculated for filgrastim and pegfilgrastim separately. Additionally, descriptive statistics were calculated for healthcare utilization, costs, and length of hospital stay during cycles with filgrastim or pegfilgrastim prophylaxis. Statistical tests were used based on the distribution of the data (eg, t-test for continuous variables and chi-squared test for categorical data). Odds ratios (OR) and 95% confidence intervals (CI) for hospitalization in cycles with pegfilgrastim prophylaxis compared to cycles with filgrastim prophylaxis were estimated by generalized estimating equation (GEE) models. The GEE models included up to the ninth chemotherapy cycle for each patient. This restriction was included because failure to do so resulted in GEE model estimation that failed to converge. A binomial distribution with log link was specified for all GEEs, and the models were fit using an exchangeable correlation structure. To control for possible confounding between G-CSF agent and outcomes, ORs were adjusted for patient age and sex, cancer type, myelotoxicity of chemotherapy, chronic comorbidities (as assessed with the Quan-Charlson comorbidity index [27]), and history of anemia in the 120 days prior to each cycle. A conventional alpha of 0.05 was used without adjustment for multiplicity. All statistical analyses were performed with SAS version 9.1 (SAS Institute Inc., Cary, NC) and Stata version 10 SE (StataCorp, College Station, TX).

### Sensitivity analysis

Sensitivity analyses were performed to determine whether other factors could affect the results. First, we examined the impact of excluding filgrastim cycles with a shorter duration (ie, less than 4 days of filgrastim prophylaxis) of filgrastim prophylaxis. Second, as the exclusion of patients with evidence of more than one primary cancer could inappropriately exclude patients with only one primary cancer (in case metastases were inappropriately coded as additional primary cancers), that criterion was removed.

## Results

### Patient characteristics

The initial population from the claims database contained 151,118 patients receiving chemotherapy. After applying the inclusion and exclusion criteria, the eligible population included 3,535 patients with breast cancer, lung cancer, NHL, ovarian cancer, or colorectal cancer receiving G-CSF prophylaxis. These patients represented 12,056 cycles during which G-CSF was delivered prophylactically, including 373 filgrastim cycles and 11,683 pegfilgrastim cycles (Table 1). Baseline demographics indicated that most patients were female with a mean age of 55, with half of the patients from the Southern region of the United States (Table 2). About half of the patients had breast cancer, with the next most frequent cancers being lung cancer (18%) and NHL (17%). Among all patient-cycles in the sample, about 32% were associated with a history of anemia in the 120 days prior to the start of each cycle. Filgrastim was used for a mean (SD) of 4.8 (3.3) injections per cycle compared to 1.0 (0.2) injections per cycle with pegfilgrastim, which was consistent with pegfilgrastim’s once-per-cycle indication.

**Table 1:** Sample selection and attrition

**Patients**	**Remaining**	
	**N**	**%**
Patients^*^	151,118	
Patients with continuous enrollment (index date minus 365 to index date)	72,978	48.3
Patients with index cancer (BC, CRC, LC, NHL, Ovarian)	18,186	24.9
Patients meeting criteria for eligible course	10,219	56.2
Patients without stem cell transplant	10,207	99.9
Patients without use of nursing facility	9,989	97.9
Patients with G-CSF use during at least one cycle	4,684	46.9
Patients without radiation on index date	4,531	96.7
Patients without more than one primary cancer site^†^	3,958	87.4
Patients with prophylactic G-CSF use in at least one cycle	3,535	89.3
Patients with prophylactic pegfilgrastim use	3,372	95.4
Patients with prophylactic filgrastim use	163	4.6
Patient-cycles with G-CSF prophylaxis in 3,535 patients	12,056	
Pegfilgrastim	11,683	96.9
Filgrastim	373	3.1
Patient-cycles with G-CSF prophylaxis [included up to the 9^th^ cycle]	11,968	
Pegfilgrastim	11,597	96.9
Filgrastim	371	3.1

BC – breast cancer, CRC – colorectal cancer, LC – lung cancer, and NHL – non-Hodgkin’s lymphoma.

^*^ Patients with any evidence of chemotherapy from January 1, 2005 to February 28, 2009 (index date set to first administration) and no chemotherapy from index date −365 to index date −1.

^†^ Primary cancers defined as: 140.xx – 172.xx, 174.xx – 195.xx and 199.xx – 208.xx.

**Table 2:** Patient characteristics

**Patient-level Characteristics**	**Total (N = 3,535)**	**Filgrastim (N = 163)**	**Pegfilgrastim (N = 3,372)**	**P-value**
Age, mean (SD)	55.2 (10.8)	57.5 (12.6)	55.1 (10.7)	0.018
Female, n (%)	2,788 (78.9)	119 (73.0)	2,669 (79.2)	0.060
Geographic region, n (%)				
Northeast	248 (7.0)	26 (16.0)	222 (6.6)	<0.001
Midwest	970 (27.4)	38 (23.3)	932 (27.6)	0.227
South	1,778 (50.3)	79 (48.5)	1,699 (50.4)	0.632
West	539 (15.3)	20 (12.3)	519 (15.4)	0.279
Tumor type, n (%)				
Breast cancer	2,054(58.1)	84 (51.5)	1,970 (58.4)	0.082
Colorectal cancer	46 (1.3)	7 (4.3)	39 (1.2)	<0.001
Lung cancer	634 (17.9)	30 (18.4)	604 (17.9)	0.873
Non-Hodgkin’s lymphoma	594 (16.8)	33 (20.3)	561 (16.6)	0.229
Ovarian cancer	207 (5.9)	9 (5.5)	198 (5.9)	0.852
Baseline Quan-Charlson comorbidity score, mean (SD)^*†^	4.5 (2.4)	4.4 (2.5)	4.5 (2.4)	0.520
Score of 2, n (%)	1,131 (32.0)	57 (35.0)	1,074 (31.9)	0.404
Score of 3–6, n (%)	1,590 (45.0)	61 (37.4)	1,529 (45.3)	0.047
Score of ≥ 7, n (%)	805 (22.8)	44 (27.0)	761 (22.6)	0.188
History of anemia^‡§^	3,858 (32.0)	135 (36.2)	3,723 (31.9)	0.078
Count of myelosuppressive chemotherapy agents, mean (SD) for cycles^§**^	2.13 (0.6)	1.96 (0.6)	2.14 (0.6)	<0.001
Use duration, mean (SD)^††^	-	4.8 (3.3)	^‡‡^	-
1 day, %	-	19.9	-	-
2 days, %	-	9.9	-	-
3 days, %	-	14.0	-	-
4–6days, %	-	23.9	-	-
≥ 7 days, %	-	32.3	-	-

^*^ Baseline is the 1-year period preceding the index date (start of chemotherapy course).

^†^ Nine patients (0.3%) had a score of 0 (7 patients) or 1 (2 patients).

^‡^ Anemia occurring in the 120 days prior to the start of each cycle.

^§^ Cycle-level data: 12,056 total cycles of which 373 were filgrastim and 11,683 were pegfilgrastim.

^**^ Data by cycle, not by patient, as myelosuppressive characteristics were determined at the cycle level.

^††^ Calculated from cycles in which filgrastim or pegfilgrastim were found on medical claims. Cycles with pharmacy claims for either were not included, as the precise date for the administration of filgrastim or pegfilgrastim could not be determined. These criteria resulted in 322 filgrastim cycles and 11,510 pegfilgrastim cycles.

^‡‡^ For pegfilgrastim, there was a mean (SD) of 1.0 (0.15) claims per cycle.

### Risk of hospitalization

Hospitalization data for a narrow definition of neutropenia, a broad definition of neutropenia, and all causes were calculated and are described herein. The incidence of neutropenia-related hospitalization (narrow definition) per cycle was 1.3% (95% CI: 0.4%–3.1%) with prophylactic filgrastim and 0.6% (95% CI: 0.5%–0.7%) with prophylactic pegfilgrastim (P = 0.063); neutropenia-related hospitalization (broad definition) per cycle also occurred at a higher incidence with prophylactic filgrastim than with prophylactic pegfilgrastim (6.7% [95% CI: 4.4%–9.7%] vs. 2.4% [95% CI: 2.1%–2.7%], P < 0.001); a higher incidence with prophylactic filgrastim than with prophylactic pegfilgrastim in all-cause hospitalizations per cycle was observed as well (10.2% [95% CI: 7.3%–13.7%] vs. 5.0% [95% CI: 4.6%–5.4%], P < 0.001).

**Figure 1:**
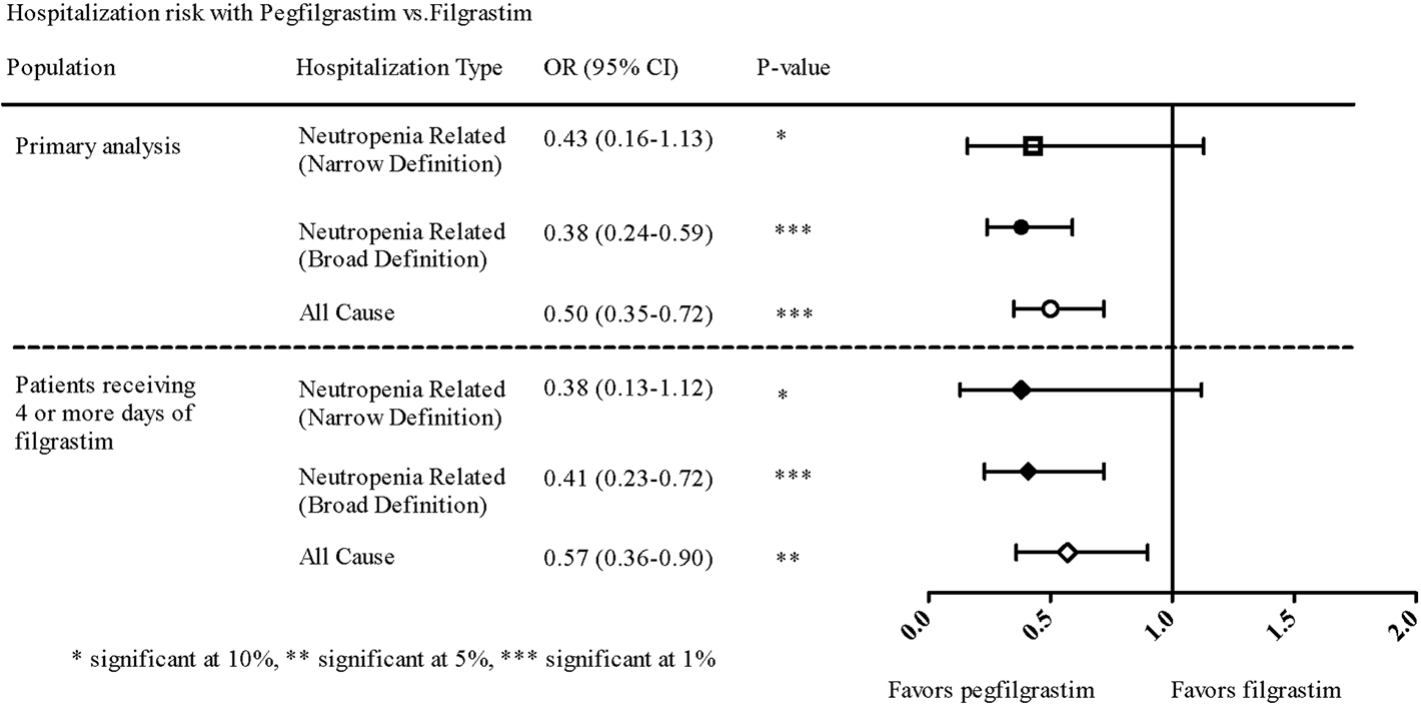
**Odds ratios for neutropenia-related and all-cause hospitalization regarding prophylactic pegfilgrastim versus prophylactic filgrastim.** Odds ratios (ORs) and 95% confidence intervals (95% CI) for hospitalization are shown with pegfilgrastim versus filgrastim prophylaxis, adjusted for patient, cancer, and chemotherapy characteristics. The primary analysis included all cycles in which G-CSF was used prophylactically. The subgroup analysis only included those cycles in which patients received 4 or more days of filgrastim (as described in the Methods section under “Sensitivity Analyses”).

When compared with cycles in which filgrastim was prophylactically used, cycles in which prophylactic pegfilgrastim was used were associated with a reduction in the risk of all-cause hospitalizations (odds ratio [OR] of 0.50, 95% CI: 0.35–0.72, P < 0.001) and neutropenia-related hospitalization (narrow definition: OR: 0.43, 95% CI: 0.16–1.13, P = 0.087; broad definition: OR: 0.38, 95% CI: 0.24-0.59, P < 0.001), after controlling for patient, disease, and treatment characteristics in GEE models (Table 3, Figure 1).

**Table 3:** Adjusted odds ratios for risk of hospitalization by G-CSF

**Variable**	**Neutropenia-Related**		**All-Cause**
	**Narrow Definition**	**Broad Definition**	
	**OR (95% CI)**	**OR (95% CI)**	**OR (95% CI)**
Prophylactic pegfilgrastim (vs. prophylactic filgrastim)	0.43 (0.16–1.13)	0.38 (0.24–0.59)	0.50 (0.35–0.72)
Age	1.01 (0.99–1.03)	1.00 (0.99–1.01)	1.00 (1.00–1.01)
Gender male	0.79 (0.39–1.59)	1.40 (1.03–1.90)	0.94 (0.94–1.18)
Baseline Quan-Charlson comorbidity score	1.02 (0.96–1.14)	1.09 (1.04–1.15)	1.07 (1.03–1.11)
Prior anemia	0.95 (0.57–1.58)	1.30 (1.02–1.66)	1.34 (1.13–1.60)
Breast cancer^*^	0.28 (0.14–0.56)	0.44 (0.32–0.61)	0.47 (0.38–0.59)
Lung cancer^*^	0.22 (0.07–0.64)	0.54 (0.37–0.77)	0.84 (0.65–1.08)
Number of myelosuppressive chemotherapy agents^†^	2.28 (1.33–3.89)	0.86 (0.68–1.07)	0.95 (0.81–1.12)

All odds ratios (OR) are adjusted for the other variables listed.

^*^ Compared to NHL, ovarian cancer, and colorectal cancer.

^†^ Myelosuppressive chemotherapy agent was defined as one of the following agents: bendamustine, busulfan, capecitabine, carboplatin, carmustine, chlorambucil, cisplatin, cladribine, clofarabine, cyclophosphamide, cytarabine, dacarbazine, daunorubicin, docetaxel, doxorubicin, epirubicin, etoposide, fludarabine, fluorouracil, hydroxyurea, idarubicin, ifosfamide, irinotecan, ixabepilone, lomustine, mechlorethamine, melphalan, mercaptopurine, methotrexate, mitoxantrone, oxaliplatin, paclitaxel, pemetrexed disodium, procarbazine, temozolomide, teniposide, thiotepa, or topotecan.

Sensitivity analyses were conducted to examine the possible effects of length of filgrastim prophylaxis and the presence of more than one primary cancer. Hospitalization risk for cycles in which filgrastim was used for 4 or more days was examined. The OR for the comparison between filgrastim and pegfilgrastim did not change appreciably from those observed in the primary analysis (Figure 1). Similar findings were observed when patients with more than one primary cancer were included in the analyses (data not shown).

### Healthcare utilization and costs

Healthcare resource utilization and costs on a per-cycle basis were examined for neutropenia-related causes (with both the broad and narrow criteria for neutropenia) and all causes (Table 4). Because similar findings were observed for both definitions of neutropenia, we only described results based on the more restrictive definition here.

**Table 4:** All-cause and neutropenia-related utilization by cycle

		**Total (N = 12,056)**	**Filgrastim (N = 373)**	**Pegfilgrastim (N = 11,683)**
All-cause	Hospitalizations n (%)^*^	620 (5.1)	38 (10.2)	582 (5.0)
	Mean ± SD^†^	0.06 ± 0.26	0.14 ± 0.46	0.05 ± 0.25
	Ambulatory visits n (%)^*^	12,051 (100)	373 (100)	11,678 (100)
	Mean ± SD^†^	5.5 ± 3.7	9.5 ± 5.9	5.4 ± 3.5
	Emergency room visits n (%)^*^	943 (7.8)	27 (7.2)	916 (7.8)
	Mean ± SD^†^	0.11 ± 0.46	0.09 ± 0.32	0.11 ± 0.46
Neutropenia- related	Hospitalizations n (%)^*^	73 (0.61)	5 (1.34)	68 (0.58)
	Mean ± SD^†^	0.01 ± 0.08	0.02 ± 0.17	0.01 ± 0.08
	Ambulatory visits n (%)^*^	3,422 (28.4)	148 (39.7)	3,274 (28.0)
	Mean ± SD^†^	0.42 ± 0.93	2.1 ± 3.3	0.36 ± 0.68
	Emergency room visits n (%)^*^	32 (0.27)	1 (0.27)	31 (0.27)
	Mean ± SD^†^	0.00 ± 0.05	0.00 ± 0.05	0.00 ± 0.05

^*^ Number of cycles with the event and percentage of those cycles among all cycles.

^†^ Mean and standard deviation of the number of events per cycle.

For neutropenia-related utilization per cycle for all cycles (including cycles without neutropenia-related utilization), there were more ambulatory visits (2.1 vs. 0.36 per cycle, P < 0.001) and hospitalizations (0.02 vs. 0.01 per cycle, P = 0.146) with filgrastim than with pegfilgrastim (mean ER visits were 0 for both groups). In the subset of filgrastim cycles in which neutropenia-related hospitalization occurred (5 cycles, 1.34% among all 373 filgrastim cycles), an average of 1.4 neutropenia-related hospitalizations occurred per cycle, with mean length of stay of 12.6 days. In the subset of pegfilgrastim cycles in which neutropenia-related hospitalization occurred (68 cycles, 0.58% among all 11,683 pegfilgrastim cycles), an average of 1.0 neutropenia-related hospitalization occurred per cycle, lasting a mean of 7.0 days each. Among all filgrastim cycles, 39.7% were associated with at least one neutropenia-related ambulatory visit; the corresponding percentage among all pegfilgrastim cycles was 28.0%. During the subset of cycles in which these neutropenia-related ambulatory visits occurred, the mean number of neutropenia-related visits was 5.3 for filgrastim cycles and 1.3 for pegfilgrastim cycles. All-cause resource utilization showed similar trends as that indicated for neutropenia-related results, and specific all-cause healthcare utilization results can be seen in Table 4.

**Figure 2:**
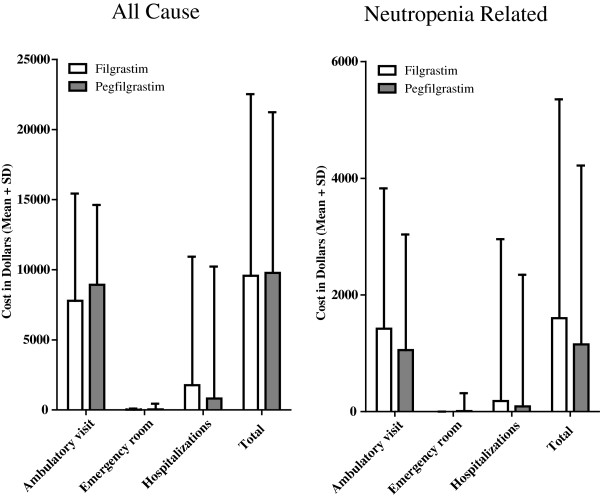
**Costs per cycle: All-cause and neutropenia-related.** Healthcare resource costs, for cycles in which pegfilgrastim and filgrastim were used prophylactically, were calculated on a per-cycle basis to provide total costs as well as subgroup costs by setting of care: ambulatory care, emergency room, and hospitalizations. The costs were direct costs for services covered under patients’ insurance benefits and represented the reimbursed amount paid by the patient and insurer.

Per-cycle costs were also examined by setting of care: ambulatory care, ER, and inpatient hospitalizations (Figure 2). Mean neutropenia-related per-cycle costs for all cycles (including cycles without neutropenia-related utilization) were greater for filgrastim than for pegfilgrastim ($1,601 vs. $1,150, P = 0.022), with the difference being driven by the greater costs of ambulatory care ($1,421 vs. $1,054, P = 0.004). The mean neutropenia-related hospitalization cost per cycle (including cycles without neutropenia-related hospitalization events) was also numerically greater for filgrastim than for pegfilgrastim ($180 vs. $88, P = 0.523). For the subset of cycles in which neutropenia-related hospitalizations occurred (as opposed to all cycles), the mean neutropenia-related hospitalization cost of $13,457 per filgrastim cycle appeared to be statistically insignificant from the $15,069 cost per pegfilgrastim cycle (P = 0.892). In the subset of cycles in which neutropenia-related ambulatory visits occurred, mean neutropenia-related ambulatory care costs per cycle with filgrastim were also similar to those for pegfilgrastim ($2,023 vs. $2,159, P = 0.413).

The combined mean costs per cycle due to all cause for all cycles were fairly similar for the two G-CSF molecules, with $9,575 for filgrastim and $9,786 for pegfilgrastim (P = 0.756). Compared to pegfilgrastim, there were lower ambulatory care costs for filgrastim ($7,788 vs. $8,933, P = 0.004) and greater hospitalization costs for filgrastim ($1,769 vs. $815, P = 0.054).

## Discussion

In this retrospective United States claims analysis, use of prophylactic pegfilgrastim was associated with a decreased risk of neutropenia-related hospitalization (narrow definition: OR = 0.43, 95% CI: 0.16–1.13; broad definition: OR = 0.38, 95% CI: 0.24–0.59) and all-cause hospitalization (OR = 0.50, 95% CI: 0.35–0.72) when compared to that seen with the use of prophylactic filgrastim. Patients who received filgrastim prophylaxis in this study had a mean 4.8 days of prophylaxis, which is much shorter than the duration which has been demonstrated to be non-inferior to pegfilgrastim prophylaxis in clinical trials [14-18]. These results are consistent with previous findings that hospitalization risk was more significantly reduced by pegfilgrastim prophylaxis than by filgrastim prophylaxis in clinical practice [20-23]. Key findings regarding utilization included an increase in neutropenia-related ambulatory visits and hospitalizations with filgrastim prophylaxis compared to pegfilgrastim prophylaxis. In addition, mean per-cycle neutropenia-related costs were greater with filgrastim prophylaxis than with pegfilgrastim prophylaxis.

In a previous study that used a large managed care claims database in the United States, Weycker et al. performed a retrospective cohort analysis of cancer patients who received filgrastim or pegfilgrastim during their first course of chemotherapy (2003**–**2005) [21]. Both all-cause (OR = 0.73, 95% CI: 0.59–0.91) and neutropenia-related (narrow definition: OR = 0.64, 95% CI: 0.41–0.99; broad definition: OR = 0.69, 95% CI: 0.52–0.92) hospitalization risk was lower with pegfilgrastim prophylaxis compared to filgrastim prophylaxis. Two subsequent claims studies reported similar results [22,23]. The consistent finding that filgrastim prophylaxis is associated with higher hospital risks than pegfilgrastim prophylaxis may be related to the less than optimal use of filgrastim in clinical practice. Specifically, while in the comparative clinical trials, filgrastim was given for an average of 10–11 days [14-18]; in the clinic, filgrastim is often given for far fewer days, resulting in suboptimal prevention of neutropenia and thus greater rates of hospitalization for neutropenic complications [19-23].

One distinct aspect of this study is that comparative filgrastim and pegfilgrastim costs and resource utilization data were reported, including details such as the number of hospitalizations, ambulatory and ER visits, and per-cycle costs. Comparative studies assessing cost and resource utilization of filgrastim and pegfilgrastim in the United States are limited. A retrospective single-time-point survey conducted by Fortner et al. assessed the human resource costs required for administering filgrastim or pegfilgrastim [28]. They concluded that a single administration with filgrastim or pegfilgrastim had equivalent human resource costs, but because of the greater number of visits required with filgrastim, the total time and human resource cost with filgrastim (14.8 hours and $364.66) in a 21-day chemotherapy cycle were more than those with pegfilgrastim (2.4 hours and $57.30).

In this study utilizing a large claims database, all-cause costs were roughly equivalent for filgrastim and pegfilgrastim on a per-cycle basis, with more spent on hospitalizations with filgrastim and more spent on ambulatory care with pegfilgrastim. It is interesting to note that although pegfilgrastim cycles had greater costs for ambulatory care, there were proportionally more ambulatory visits for filgrastim cycles. The increased all-cause per-cycle cost of pegfilgrastim ambulatory care may reflect the greater drug costs of pegfilgrastim compared to filgrastim, especially when fewer than the recommended number of filgrastim doses were administered. Neutropenia-related costs were greater for filgrastim than pegfilgrastim because of the greater costs of both inpatient and ambulatory care during filgrastim cycles. Overall, these data suggest that the greater drug costs with pegfilgrastim are offset by decreased hospitalization costs.

There are several sources of bias and limitations inherent in the study design that could influence interpretation of these results. As this was a database of a large employment-sponsored managed care population, patients would most likely be in the 18–64 year age range. Thus, the effects of G-CSF on outcomes in the population of patients aged 65 years or above were not fully captured. Additionally, the data are dependent on the accuracy of claims coding and hence contain any errors or omissions that occurred during that coding. By using the broad criteria for neutropenia, as well as all-cause utilization, we were able to more accurately provide upper limits for our estimates. It should be noted that this study compared risk between treatment cohorts. The potential under-coding and mistakes in coding of febrile neutropenia are unlikely to be associated with G-CSF selection and thus do not affect the estimates of interest (ie, ORs). Sample size is another issue that affects the statistical power of our estimates, as there were fewer than 400 cycles with prophylactic filgrastim use. Another source of bias is the assumption that G-CSF administration by day 5 of a cycle represents prophylaxis rather than treatment. Although this definition has been used in other studies [19,21], its validity has not yet been confirmed in the literature. Thus, it is uncertain whether earlier or later onset of administration may represent prophylaxis or treatment in a clinical setting. Likewise, our categorization of certain cycles as containing highly myelosuppressive chemotherapy based on the presence of individual agents used in that cycle may not adequately capture the various factors that affect the myelosuppressive effects of a chemotherapy regimen, such as combination chemotherapy and doses of specific agents. Furthermore, potential differences across health plans covered in the study sample were not adjusted for comparison of costs between filgrastim and pegfilgrastim cycles.

It is unclear whether this study adequately captured the various known patient, disease, and treatment characteristics that are risk factors for developing febrile neutropenia [6,12]. The claims database contained several of these, such as age, sex, comorbidities, recent history of anemia, history of radiation, tumor type, and number of myelosuppressive agents. To reduce the effect of possible selection bias, data were adjusted for those covariates in the GEE model. However, the claims database did not include other potential predictors of febrile neutropenia, such as treatment intent, disease stage, chemotherapy dose, previous febrile neutropenia events, laboratory values, and concomitant medications. Some of these factors could influence selection of G-CSF as either filgrastim or pegfilgrastim. Thus the study results may still be confounded by possible differences in those unobserved characteristics between filgrastim and pegfilgrastim groups.

The use of per-cycle analyses for utilization and costs has the disadvantage of not capturing costs associated with cycles in which G-CSF was not administered. However, per-patient analyses would not allow us to temporally associate use of either G-CSF with utilization. For instance, in a per-patient analysis, a patient hospitalized in cycle 1 who subsequently received G-CSF in cycle 2 would have those two events associated, when clearly the hospitalization was independent of any effects from G-CSF. Another disadvantage of per-patient analyses is that while on average, each patient who received filgrastim received it for a mean of 2.3 cycles, while each patient who received pegfilgrastim received it for a mean of 3.5 cycles. The accompanying drug costs would make it difficult to discern any possible effect of either medication on all-cause medical costs.

In conclusion, the results of this claims analysis indicate that prophylactic use of filgrastim as compared with pegfilgrastim is associated with an increased risk of hospitalization from all causes as well as neutropenia-related causes. Results from this analysis and others indicate that other factors influence the risk of hospitalization, including comorbidities, history of anemia, age, and presence of metastatic disease [2,6,11,12,29]. Future studies that explore the role of these characteristics will help further clarify the various factors that lead to febrile neutropenia and associated complications.

## Conclusions

This retrospective comparative effectiveness study used claims data to examine prophylactic use of filgrastim and pegfilgrastim in cancer patients receiving chemotherapy. The key finding of the study is that pegfilgrastim prophylaxis was associated with a reduced risk of hospitalization due to neutropenia or all-cause.

## Competing interests

In addition to the relationships with Amgen Inc. described in the Acknowledgements, Henry Henk and Laura Becker are employees of OptumInsight, while Victoria Chia, Sejal Badre, Xiaoyan Li, and Robert Deeter are stockholders of Amgen Inc.

## Authors’ contributions

All authors contributed to study design and analysis and interpretation of data. HH and LB were primarily responsible for data acquisition. All authors read and approved the final manuscript.

## Pre-publication history

The pre-publication history for this paper can be accessed here:

http://www.biomedcentral.com/1471-2407/13/11/prepub

## Supplementary Material

Additional file 1 Appendix - Bacterial and Fungal Infections. Click here for file
