# Supplementary material for: Pegfilgrastim prophylaxis is associated with a lower risk of hospitalization of cancer patients than filgrastim prophylaxis: a retrospective United States claims analysis of granulocyte colony-stimulating factors (G-CSF)
Source: BMC Cancer. 2013 Jan 8;13:11. doi: 10.1186/1471-2407-13-11 (PMC3559272; doi:10.1186/1471-2407-13-11)
Supplement: Additional file 1 — Appendix - Bacterial and Fungal Infections. [file 1471-2407-13-11-S1.docx]

Appendix - Bacterial and Fungal Infections

| ICD-9-CM code | Description |
| --- | --- |
| 001 | Cholera |
| 002 | Typhoid and paratyphoid fevers |
| 003 | Other salmonella infections |
| 004 | Shigellosis |
| 005 | Other food poisoning |
| 008.0 | Intestinal infections due to E. coli |
| 008.1 | Intestinal infections due to Arizona group of paracolon bacilli |
| 008.2 | Intestinal infections due to Aerobacter aerogenes |
| 008.3 | Intestinal infections due to Proteus (mirabilis) (morganii) |
| 008.4 | Intestinal infections due to other specified bacteria |
| 008.5 | Bacterial enteritis, unspecified |
| 009 | Ill-defined intestinal infections |
| 013 | CNS tuberculosis |
| 018 | Miliary tuberculosis |
| 020 | Plague |
| 021 | Tularemia |
| 022 | Anthrax |
| 023 | Brucellosis |
| 024 | Glanders |
| 025 | Melioidosis |
| 026 | Rat-bite fever |
| 027 | Other bacterial zoonoses |
| 032 | Diphtheria |
| 033 | Whooping cough |
| 034 | Streptococcal throat/scarlet fever |
| 035 | Erysipelas |
| 036 | Meningococcal infection |
| 037 | Tetanus |
| 038 | Septicemia |
| 039 | Actinomycotic infections |
| 040 | Other bacterial diseases |
| 041 | Bacterial infection in other diseases not specified |
| 098 | Gonococcal infections |
| 100 | Leptospirosis |
| 101 | Vincent's angina |
| 112.0 | Candidiasis, of mouth |
| 112.4 | Candidiasis, of lung |
| 112.5 | Candidiasis, disseminated |
| 112.8 | Candidiasis, of other specified sites |
| 114 | Coccidioidomycosis |
| 115 | Histoplasmosis |
| 116 | Blastomycotic infection |
| 117 | Other mycoses |
| 118 | Opportunistic mycoses |
| 320 | Bacterial meningitis |
| 321.0 | Cryptococcal meningitis |
| 321.1 | Meningitis in other fungal diseases |
| 324 | CNS abscess |
| 325 | Phlebitis of intracranial sinus |
| 360.0 | Purulent endophthalmitis |
| 376.0 | Acute inflammation of orbit |
| 380.14 | Malignant otitis externa |
| 383.0 | Acute mastoiditis |
| 420.99 | Acute pericarditis due to other specified organisms |
| 421 | Acute and subacute endocarditis |
| 461 | Acute sinusitis |
| 462 | Acute pharyngitis |
| 463 | Acute tonsillitis |
| 464 | Acute laryngitis and tracheitis |
| 465 | Acute upper respiratory infections of multiple sites/not otherwise specified |
| 475 | Peritonsillar abscess |
| 481 | Pneumococcal pneumonia |
| 482 | Other bacterial pneumonia |
| 485 | Bronchopneumonia, organism unspecified |
| 486 | Pneumonia, organism unspecified |
| 491.21 | Obstructive chronic bronchitis with acute exacerbation |
| 494 | Bronchiectasis |
| 510 | Empyema |
| 513 | Abscess of lung and mediastinum |
| 522.5 | Periapical abscess without sinus |
| 522.7 | Periapical abscess with sinus |
| 526.4 | Inflammatory conditions of the jaw |
| 527.3 | Abscess of the salivary glands |
| 528.3 | Cellulitis and abscess of oral soft tissues |
| 540 | Acute appendicitis |
| 541 | Appendicitis, not otherwise specified |
| 542 | Other appendicitis |
| 562.01 | Diverticulitis of small intestine without hemorrhage |
| 562.03 | Diverticulitis of small intestine with hemorrhage |
| 562.11 | Diverticulitis of colon without hemorrhage |
| 562.13 | Diverticulitis of colon with hemorrhage |
| 566 | Abscess of anal and rectal regions |
| 567 | Peritonitis |
| 569.5 | Intestinal abscess |
| 569.61 | Infection of colostomy or enterostomy |
| 569.83 | Perforation of intestine |
| 572.0 | Abscess of liver |
| 572.1 | Portal pyemia |
| 575.0 | Acute cholecystitis |
| 590 | Kidney infection |
| 599.0 | Urinary tract infection, not otherwise specified |
| 601 | Prostatic inflammation |
| 604 | Orchitis and epididymitis |
| 614 | Female pelvic inflammation disease |
| 615 | Uterine inflammatory disease |
| 616.3 | Abscess of Bartholin's gland |
| 616.4 | Other abscess of vulva |
| 646.6 | Infections of genitourinary tract in pregnancy |
| 658.4 | Infection of amniotic cavity |
| 670 | Major puerperal infection |
| 675.1 | Abscess of breast |
| 681 | Cellulitis, finger/toe |
| 682 | Other cellulitis and abscess |
| 683 | Acute lymphadenitis |
| 685.0 | Pilonidal cyst with abscess |
| 686 | Other local skin infection |
| 711.0 | Pyogenic arthritis |
| 728.86 | Necrotizing fasciitis |
| 730 | Osteomyelitis |
| 790.7 | Bacteremia |
| 958.3 | Posttraumatic wound infection, not elsewhere classified |
| 996.6 | Infection or inflammation of device/graft |
| 998.5 | Postoperative infection |
| 999.3 | Infectious complications of medical care not otherwise specified |
